# Role of GP empathy on patient-reported outcomes in telephone consultations: a cross-sectional study with validation of the Consultation and Relational Empathy (CARE) Measure

**DOI:** 10.3399/BJGP.2025.0475

**Published:** 2026-04-08

**Authors:** Kieran D Sweeney, Lauren Ng, Brian Kelly, Diana Stamatopoulos, Jeremy Howick, Stewart W Mercer

**Affiliations:** 1 Usher Institute, College of Medicine and Veterinary Medicine, University of Edinburgh, Edinburgh, UK; 2 College of Medicine and Veterinary Medicine, University of Edinburgh, Edinburgh, UK; 3 Stoneygate Centre for Empathic Healthcare, University of Leicester, Leicester, UK

**Keywords:** cross-sectional studies, empathy, general practice, patient outcomes, primary health care, remote consultation

## Abstract

**Background:**

The Consultation and Relational Empathy (CARE) Measure is a validated measure of GP empathy in face-to-face consultations (FTFCs) and higher CARE scores predict better patient outcomes. However, the validity of the CARE Measure and its importance in telephone consultations (TCs) is unknown.

**Aim:**

To determine the validity and reliability of the CARE Measure in TCs, and the associations between CARE scores and outcomes in TCs compared with FTFCs.

**Design and setting:**

A cross-sectional survey, conducted in 2018, of 1023 patients who had a TC or FTFC with a GP in Scotland within the preceding 30 days.

**Method:**

Validity and reliability testing of the CARE Measure was conducted using standard methods. Associations between low, medium, and high CARE scores and three patient outcomes (enablement, symptom change, and satisfaction) were determined using multilevel binary logistic regression.

**Results:**

Out of the 1023 participants, 369 had TCs and 654 had FTFCs. The CARE Measure was found to be valid and reliable. In TCs, compared with the low CARE score group, a high CARE score was positively associated with enablement (adjusted odds ratio [aOR] 6.4, 95% confidence interval [CI] = 3.5 to 11.9), symptom improvement (aOR 5.7, 95% CI = 2.7 to 11.9), and satisfaction (aOR 20.1, 95% CI = 8.9 to 45.4). Findings were similar in FTFCs,and effects were not influenced by patient or consultation characteristics in either group.

**Conclusion:**

The CARE Measure is valid and reliable in TC. GP empathy, as measured by the tool, predicts better patient outcomes in TCs, similar to FTFCs. Given the common use of TC in primary care, strategies to support empathic communication are essential irrespective of consultation modality.

## How this fits in

Clinician empathy is known to improve a range of outcomes in face-to-face primary care consultations, but there is limited evidence regarding its impact in telephone consultations (TCs). This study showed that GP empathy (measured with the patient-rated CARE [Consultation and Relational Empathy] Measure, shown to be valid in both TCs and face-to-face consultations [FTFCs]) is associated with better patient enablement, satisfaction, and symptom improvement in both TCs and FTFCs. The association between GP empathy and patient-reported outcomes was found to be equally strong in TCs as in FTFCs, irrespective of patient and consultation characteristics. Given the common use of TC in primary care, strategies to support empathic communication are essential, irrespective of consultation modality.

## Introduction

The use of telephone consultations (TCs) in primary care increased rapidly during the COVID-19 pandemic,^1^ and has since become a routine part of general practice in many high-income countries. In the UK, approximately 30% of GP consultations are now conducted by telephone, compared with just 10% before the pandemic.^2–4^

TCs may offer a more time-efficient alternative to face-to-face consultations (FTFCs) for clinicians, and have been seen as a response to the growing pressures in primary care.^5,6^ For some patients, they are also seen as more convenient and accessible.^7,8^ While some evidence suggests that TC can be as clinically effective as FTFC,^5^ concerns remain about their quality and safety.^6^ Studies have reported lower patient satisfaction and a higher risk of diagnostic error in TC compared with FTFC.^6,9^ Moreover, disparities have been observed in their uptake, with TC used more frequently with patients living in socioeconomically deprived areas despite the higher prevalence of complex problems.^10–12^

Physician empathy is a key component of high-quality care and is consistently associated with improved patient outcomes, including greater satisfaction, increased patient enablement, and symptom improvement.^13–16^ However, most existing research on empathy and its effects on patient outcomes has focused on FTFC. Less is known about how empathic care operates, and whether it influences outcomes, within the context of TC. The Consultation and Relational Empathy (CARE) Measure is a widely used instrument to evaluate a patient’s perception of physician empathy, which has been validated in FTFC.^17^ However, to the authors’ knowledge, it has not been validated in TC.

The aims of the current study were to determine the validity and reliability of CARE in TCs, and to determine the associations between CARE and outcomes in TC compared with FTFC.

## Method

### Study design

This cross-sectional study analysed data from a postal survey, conducted as part of a broader evaluation of the 2018 Scottish GP contract.

### Sampling, recruitment, and data collection

Full methodological details have been published previously.^12^ In brief, 12 general practices across three health board regions in Scotland were purposefully selected to represent a range of geographic and socioeconomic characteristics, with four practices selected per region. Within each practice, a random sample of adults (aged ≥18 years) who had consulted a GP within the preceding 30 days was identified from practice records. A total of 6291 postal surveys were distributed, with a response rate of 17% (*n* = 1053). This secondary analysis study focused on responders who had either a TC or FTFC (*n* = 1023). Patients who received home visits (*n* = 11) or video consultations (*n* = 1), and those with missing responses for consultation modality (*n* = 18), were excluded from analysis.

### Instruments

The survey collected data on GP empathy, consultation modality, patient-reported outcomes, and relevant covariates. The primary explanatory variable was patient-perceived GP empathy, measured using the 10-item CARE Measure.^17^ The grouping variable was consultation modality, self-reported by patients as either a TC or FTFC.

Outcomes comprised three patient-reported measures:

- patient enablement, measured using the Patient Enablement Instrument (PEI);^18^
- patient satisfaction, rated on a 7-point Likert scale; and
- symptom improvement, measured using the Outcome in Relation to Impact on Daily Living (ORIDL) scale.^15^

Demographic variables included age, sex, living arrangement, socioeconomic deprivation (measured from each patient’s postcode using the Scottish Index of Multiple Deprivation),^19^ and geographic region.^20^ Health characteristics included self-rated health, disability status, multimorbidity (defined as the presence of ≥2 long-term conditions), symptoms of depression and anxiety,^21^ and consultation frequency. Consultation characteristics included the number of problems discussed, time since consultation (in weeks), and problem complexity (a binary variable based on whether the problem discussed in the consultation was both physical and psychosocial in nature).^12^

### Analysis

Data analysis was conducted using SPSS Statistics (version 27) and R (version 4.5.0). The validity and reliability of the CARE Measure in TC and FTFC was determined in several ways. Face and construct validity was explored based on the number of missing items, and the number of ‘not applicable’ responses to each item, as in previous work. Internal reliability was measured by Cronbach’s alpha overall, and the effect of removing each item in turn on the alpha value (a decrease in alpha indicates that the item is contributing to the scale’s reliability). Corrected item-total correlations were used to examine how well each CARE Measure item correlated with the sum of scores on all other items in the scale, excluding the item itself, which indicates the item’s contribution to the overall scale’s reliability and validity. High correlations indicate that an item is a good indicator of the construct the scale is aiming to measure. Factor analysis was used to investigate whether TC CARE response resulted in a single factor solution (based on an eigenvalue >1), as found in numerous previous studies on the CARE Measure in FTFCs. Before conducting factor analysis, the authors checked that the Kaiser–Meyer–Olkin (KMO) value was >0.5 (indicating an adequate sample size) and that the Bartlett test of sphericity was significant (*P*<0.05), which indicated that the variables were correlated and suitable for factor analysis.

Binary logistic regression was used to examine the association between patient-perceived GP empathy and each patient-reported outcome (patient satisfaction, enablement, and symptom improvement) by consultation modality. Because of non-normal distributions of the three outcome measures (Supplementary Figure S1), the outcomes were dichotomised using established thresholds based on previous work.^15,22^ Specifically, these were:

- patient satisfaction was categorised as <6 or ≥6 on a 7-point Likert scale;
- symptom improvement (ORIDL) was categorised as 0–1 versus 2–3; and
- patient enablement (PEI) was categorised as ≤3 versus >3.

To improve interpretability, CARE Measure scores (with a possible range 10–50) were transformed into a three-level ordinal variable (low, moderate, and high empathy) based on tertiles. A multilevel model was used given the hierarchical nature of the data, with adjustment for GP and GP practice as random effects. This was supported by examination of intraclass correlation coefficients (ICC) for each outcome (Supplementary Table S1). Health board level was not included as the researchers’ statistician (third author) advised that a sample size of three was too small for multilevel modelling, and in any case the ICC for each outcome was zero, and there were no significant differences in outcomes between the three health board (data not shown). Potentially relevant covariates were identified from previous work.^17,22,23^ To determine which covariate to include in the final model, each covariate was examined in turn by inclusion in a basic model along with age, sex, and deprivation, plus random effects for GP and practice. Covariates that demonstrated a significant association (*P*<0.1) for each outcome in this basic model were then included in the final model ( Supplementary Table S2). This resulted in the inclusion of the following six fixed effects in the model for all three outcomes: age, sex, deprivation, self-rating of health, disability, and the presence of anxiety/depression symptoms. For two outcomes (symptom improvement and satisfaction) problem complexity was also included. The authors also tested for interactions between the primary explanatory variable (CARE score) and the following: deprivation, depression/anxiety, disability, and problem complexity.

## Results

Participating patients’ characteristics in TC (*n* = 369) and FTFC (*n* = 654) consultations are shown in Table 1. TCs were more common among female patients and in patients who had consulted more frequently in the previous 12 months. Statistically, patients seen face-to-face discussed significantly more problems per consultation than those receiving TCs. There were no statistically significant differences between the two consultation modalities for the remaining demographic, health, or consultation variables.

**Table 1.** Patient demographic, health, and consultation characteristics in telephone and face-to-face consultations

Characteristic	Telephone, *n* = 369	Face-to-face, *n* = 654	*P*-value
**Sex, *n* (%)**			0.002
Male	121 (32.8)	275 (42.0)	
Female	237 (64.2)	355 (54.3)	
Missing	11 (3.0)	24 (3.7)	
**Age, years, *n* (%)**			0.535
<45	44 (11.9)	94 (14.4)	
45–64	132 (35.8)	221 (33.8)	
≥65	183 (49.6)	327 (50.0)	
Missing	10 (2.7)	12 (1.8)	
Mean (SD)	62.9 (15.3)	62.3 (15.9)	
**Living arrangement*, n* (%)**			0.249
With someone	248 (67.2)	468 (71.6)	
Alone	109 (29.5)	174 (26.6)	
Missing	12 (3.3)	12 (1.8)	
**Deprivation quintile, *n* (%)**			0.222
1 (most deprived)	81 (22.0)	107 (16.4)	
2	83 (22.5)	158 (24.2)	
3	63 (17.1)	148 (22.6)	
4	53 (14.4)	65 (9.9)	
5 (least deprived)	89 (24.1)	175 (26.8)	
Missing	0 (0)	1 (0.2)	
**Self-rating of health^a^**			0.073
Mean (SD)	3.31 (0.996)	3.43 (1.02)	
Missing, *n* (%)	3 (0.8)	2 (0.3)	
**Disability, *n* (%)**			0.191
No	191 (51.8)	369 (56.4)	
Yes	171 (46.3)	278 (42.5)	
Missing	7 (1.9)	7 (1.1)	
**Multimorbidity, *n* (%)**			0.642
No (0–1 LTC)	111 (30.1)	207 (31.7)	
Yes (≥2 LTC)	256 (69.4)	447 (68.3)	
Missing	2 (0.5)	0 (0.0)	
**Depression and anxiety symptoms**			0.123
Mean (SD)	2.93 (3.44)	2.70 (3.44)	
Missing, *n* (%)	7 (1.9)	11 (1.7)	
**Consultation frequency^b^**			0.005
Mean (SD)	5.08 (4.13)	4.36 (3.49)	
Missing, *n* (%)	16 (4.3)	18 (2.8)	
**Number of problems discussed**			<0.001
Mean (SD)	1.43 (0.616)	1.68 (0.729)	
Missing, *n* (%)	24 (6.5)	57 (8.7)	
**Complex problem discussed, *n* (%)**			0.762
No	317 (85.9)	556 (85.0)	
Yes	44 (11.9)	82 (12.5)	
Missing	8 (2.2)	16 (2.4)	

^a^Measured on scale 1–5 (1 = excellent; 5 = poor). ^b^Number of GP consultations over the past 12 months. LTC = long-term condition. SD = standard deviation.

### Validity and reliability of the CARE Measure in telephone and face-to-face consultations

Responses to all of the CARE Measure items were negatively skewed with a ceiling effect in both consultation modalities (Supplementary Table S3 and Figure S2). The frequency of missing values across all 10 items was extremely low (<2%) in both TC and FTFC, suggesting a high face validity of the CARE measure (Table 2). Content validity was supported by the low number of ‘not applicable’ responses, though these were slightly higher in TCs (average 7.9%) than FTFCs (average 3.9%). The highest frequency of ‘not applicable’ responses were for items 9 and 10 in both consultation modalities, whereas item 3 had a substantially higher frequency of ‘not applicable’ responses for TCs than for FTFC (13.8% versus 0.8%, respectively).

**Table 2.** Validity and reliability of the CARE Measure in telephone (*n* = 369) and face-to-face consultations (*n* = 654)

	Missing, *n* (%)		‘Not applicable’, *n* (%)		Cronbach’s alpha (item dropped)		Corrected item-total correlation		Factor loadings	
**CARE Measure item number**	**TC**	**FTFC**	**TC**	**FTFC**	**TC**	**FTFC**	**TC**	**FTFC**	**TC**	**FTFC**
1	2 (0.5)	3 (0.5)	8 (2.2)	1 (0.2)	0.982	0.977	0.879	0.841	0.903	0.872
2	2 (0.5)	4 (0.6)	17 (4.6)	2 (0.5)	0.981	0.976	0.906	0.883	0.926	0.907
3	3 (0.8)	3 (0.5)	51 (13.8)	5 (0.8)	0.980	0.975	0.932	0.904	0.947	0.924
4	3 (0.8)	7 (1.1)	30 (8.1)	28 (4.2)	0.981	0.975	0.901	0.903	0.920	0.922
5	2 (0.5)	4 (0.6)	17 (4.6)	9 (1.4)	0.980	0.974	0.936	0.933	0.949	0.947
6	4 (1.1)	5 (0.8)	15 (4.1)	9 (1.4)	0.980	0.975	0.945	0.912	0.956	0.930
7	2 (0.5)	6 (0.9)	19 (5.1)	12 (1.8)	0980	0.975	0.931	0.908	0.944	0.925
8	2 (0.5)	7 (1.1)	22 (5.9)	8 (1.2)	0.980	0.976	0.930	0.893	0.943	0.913
9	5 (1.4)	8 (1.2)	61 (16.5)	101 (15.2)	0.981	0.976	0.930	0.896	0.923	0.915
10	2 (0.5)	8 (1.2)	51 (13.8)	79 (11.9)	0.982	0.977	0.882	0.869	0.903	0.983
Total	27	55	291	254	0.983	0.978	—	—	—	—

FTFC = face-to-face consultation. TC = telephone consultation.

The CARE Measure demonstrated high internal reliability in both TC and FTFC, with a Cronbach’s alpha of 0.983 and 0.978, respectively (Table 2). Removal of any of the 10 items in both consultation modalities reduced the Cronbach’s alpha. Corrected item-total correlations were also high in both consultation modalities. KMO was 0.956 and 0.958 for TC and FTFC, respectively, indicating a sufficient sample size for factor analysis, and Bartlett’s test was highly significant (*P*<0.001) for both consultation modalities, indicating sufficient correlation between the CARE Measure items to warrant factor analysis (data not shown). Factor analysis gave a single-factor solution in both TC and FTFC, which explained 86.8% and 83.8% of variance, respectively. High factor loadings were found for both TC and FTFC, supporting construct validity (Table 2).

### Association between GP empathy and patient-reported outcomes

Table 3 presents the unadjusted (ORs) and adjusted odds ratios (aORs) for the association between GP empathy (CARE score in tertiles) and each patient-reported outcome (enablement, satisfaction, and symptom improvement), stratified by consultation modality (see Supplementary Table S4 for the regression models in full). In TC, there were no significant interaction effects between empathy and the patient and consultation characteristics, and for FTFC only one significant interaction with empathy was found, for disability and patient satisfaction (data not shown).

**Table 3.** Association between GP empathy (CARE score) and patient-reported outcomes in telephone and face-to-face consultations

Consultation mode	CARE score level (reference: low)	Unadjusted OR (95% CI)	Adjusted OR (95% CI)	*P*-value
**Outcome: patient enablement**				
Face-to-face	Moderate	3.09 (2.04 to 4.72)	2.93 (1.87 to 4.58)	<0.001
	High	5.74 (3.77 to 8.87)	6.21 (3.90 to 9.89)	<0.001
Telephone	Moderate	2.66 (1.51 to 4.74)	2.92 (1.57 to 5.45)	<0.001
	High	6.12 (3.50 to 10.97)	6.42 (3.48 to 11.84)	<0.001
**Outcome: patient satisfaction**				
Face-to-face	Moderate	8.31 (5.36 to 13.10)	9.09 (5.48 to 15.09)	<0.001
	High	29.23 (16.25 to 56.29)	36.29 (17.85 to 73.78)	<0.001
Telephone	Moderate	12.24 (6.45 to 24.53)	11.59 (5.61 to 23.94)	<0.001
	High	21.62 (10.63 to 48.23)	20.12 (8.93 to 45.35)	<0.001
**Outcome: symptom improvement**				
Face-to-face	Moderate	2.89 (1.84 to 4.54)	2.60 (1.61 to 4.22)	<0.001
	High	4.49 (2.78 to 7.27)	4.26 (2.53 to 7.16)	<0.001
Telephone	Moderate	2.76 (1.47 to 5.27)	3.06 (1.49 to 6.31)	<0.001
	High	4.44 (2.38 to 8.49)	5.68 (2.70 to 11.93)	<0.001

OR = odds ratio.

Consultations with moderate and high GP empathy (compared with low empathy) for patient enablement were associated with incrementally greater odds of a positive outcome in unadjusted and adjusted analyses, with comparable findings in both TC and FTFC (TC: moderate empathy aOR 2.9, 95% confidence interval [CI] = 1.6 to 5.5 and high empathy aOR 6.4, 95% CI = 3.5 to 11.8; FTFC: moderate empathy aOR 2.9, 95% CI = 1.9 to 4.6 and high empathy aOR 6.2, 95% CI = 3.9 to 9.9). GP empathy remained a significant predictor for symptom improvement, with comparable aORs across both consultation modalities (TC: moderate empathy aOR 3.1, 95% CI = 1.5 to 6.3 and high empathy aOR 5.7, 95% CI = 2.7 to 11.9; FTFC: moderate empathy aOR 2.6, 95% CI = 1.6 to 4.2 and high empathy aOR 4.3, 95% CI = 2.5 to 7.2). Associations with GP empathy were strongest for patient satisfaction, with aORs of 11.6 (95% CI = 5.6 to 23.9) for moderate empathy and 20.1 (95% CI = 8.9 to 45.4) for high empathy in TC. In FTFC, aORs were 9.1 (95% CI = 5.5 to 15.1) for moderate empathy and 36.3 (95% CI = 17.9 to 73.8) for high empathy. For all three outcomes, ORs and aORs were similar in TC and FTFC, with overlapping CIs (Table 3).

## Discussion

### Summary

This study examined the association between perceived GP empathy and patient-reported outcomes (enablement, satisfaction, and symptom improvement) across TC and FTFC in primary care. The validity and reliability of the CARE Measure in assessing patients’ perceptions of GP empathy in TC was confirmed, and was similar to FTFC. In both unadjusted and adjusted multilevel regression analyses, higher perceived GP empathy was significantly associated with better patient enablement, higher consultation satisfaction, and greater symptom improvement. There were no significant interactions between key patient characteristics or consultation complexity on the effect of GP empathy on outcomes in TC. For all three outcomes, the strength of association with GP empathy was similar in TC and FTFC.

Higher levels of empathy in TC benefit patients as much as higher levels of empathy in FTFC. With an increasingly digital primary care landscape, embedding strategies to support empathic communication in remote consultations will be critical to maintaining high-quality, equitable care irrespective of consultation modality.

### Strengths and limitations

This study’s strengths include a sample size of >1000 responders and the use of validated instruments. Additionally, adjustments of clustering effects and relevant patient and consultation variables by multilevel multiregression analysis strengthen the robustness of the observed associations.

Limitations include a modest response rate (17%), though this aligns with other UK postal surveys,^3^ and the authors have shown in a previous article using this same dataset that the responders to the survey were broadly representative of the patients invited to participate (all adult patients who had consulted a GP in the last 4 weeks in the 12 practices that took part in the study across three health boards), though responders were slightly older and in the most deprived health board were slightly less deprived than non-responders.^12^ However, the key aim of the current study, which was to determine the importance of empathy in the three patient-reported outcomes, was not dependent on a perfectly representative sample of the consulting patients, given that the authors controlled for potential confounding variables, including age and deprivation, in the multilevel multiregression analyses. Having a wide spread of patient characteristics (including deprivation and age) in the current sample was sufficient to ensure robustness of findings. However, the sample sizes may have been inadequate to identify significant interaction effects between some covariates and empathy on outcomes in the current TC analysis, and further work on a larger sample is required to confirm the lack of interaction effects.

### Comparison with existing literature

The relationship between clinician empathy and improved patient outcomes in FTFCs is well established,^14,23–26^ with benefits spanning mental health, self-management, medication adherence, quality of life, and long-term health outcomes — aligning with the results from this study. This study also extends these outcome findings to TC, which has previously been underexplored.

The current findings on the validity and reliability of the CARE Measure in consultation modalities, including the relatively high percentage of ‘not applicable’ responses for items 9 (‘helping you take control’) and 10 (‘making a plan of action’), is in agreement with previous work on the measure in face-to-face encounters. The likely reason for this is that not all consultations require self-management support, nor formulation of a plan of action, for example, a follow-up consultation. In TCs, item 3 (‘really listening’) had a much higher frequency of ‘not applicable’ responses than FTFC. This could be because patients assumed that in a TC the doctor would be listening carefully, or possibly because patients were not able to judge this because of a lack of visual non-verbal cues. Further qualitative work is required to explore this, and it may mean that, for TCs, modification or removal of item 3 may be warranted.

### Implications for research and practice

This study highlights that GP empathy remains a key predictor of patient-reported outcomes, even in TC. Importantly, when empathy is effectively conveyed, TC can deliver outcomes comparable to face-to-face care. Some studies have reported lower perceived empathy in TC compared with FTFC, including the authors’ previous article using the current dataset, which found lower TC empathy scores from patients living in remote and rural areas,^12^ and it has been suggested that the absence of visual and non-verbal cues may hinder empathic connection.^9,26,27^ However, prior work shows that empathic communication can still be effectively delivered in remote settings using intentional empathic verbalisations.^8,28^

In light of Scotland’s Digital Health and Care Strategy^29^ and the NHS England 10-Year Health Plan,^30^ which aims to offer all patients the right to have digital-first primary care, together with the global trend towards more routine TC in primary care, ensuring their quality and safety is an important challenge.^6,9^ Future research should explore how empathy can be effectively fostered within TC, as well as other digital consultation formats, for example, text, video, and artificial intelligence-assisted. Future research should also investigate which verbal strategies (such as summarising or verbal mirroring) are most effective, and for whom, and which behaviours need to be fostered in different contexts (TC, FTF, and other digital).

These findings are particularly relevant for practices in socioeconomically deprived areas, where TCs are more frequently used,^12^ and where patients often have more complex health and social needs.^31,32^ Supporting empathic care in TC may therefore also be valuable in mitigating health inequalities.

## Data

The authors do not have ethical permission or patient consent to share the full data.

## References

[1] Murphy M, Scott LJ, Salisbury C (2021). Implementation of remote consulting in UK primary care following the COVID-19 pandemic: a mixed-methods longitudinal study. Br J Gen Pract.

[2] NHS England (2026). GP Patient Survey: survey results and materials.

[3] Scottish Government (2024). Health and care experience survey.

[4] Royal College of General Practitioners (2025). Key general practice statistics and insights. https://www.rcgp.org.uk/representing-you/key-statistics-insights#appointments.

[5] Carrillo de Albornoz S, Sia K-L, Harris A (2022). The effectiveness of teleconsultations in primary care: systematic review. Fam Pract.

[6] Payne R, Clarke A, Swann N (2024). Patient safety in remote primary care encounters: multimethod qualitative study combining Safety I and Safety II analysis. BMJ Qual Saf.

[7] Scottish Government (2022). Scottish Social Attitudes Survey 2021/22: public views of telephone and video appointments in general practice. https://www.gov.scot/publications/scottish-social-attitudes-survey-2021-22-public-views-telephone-video-appointments-general-practice.

[8] Vennik J, Hughes S, Lyness E (2023). Patient perceptions of empathy in primary care telephone consultations: a mixed methods study. Patient Educ Couns.

[9] Hammersley V, Donaghy E, Parker R (2019). Comparing the content and quality of video, telephone, and face-to-face consultations: a non-randomised, quasi-experimental, exploratory study in UK primary care. Br J Gen Pract.

[10] Parker RF, Figures EL, Paddison CA (2021). Inequalities in general practice remote consultations: a systematic review. BJGP Open.

[11] Vestesson EM, De Corte KLA, Crellin E (2023). Consultation rate and mode by deprivation in English general practice from 2018 to 2022: population-based study. JMIR Public Health Surveill.

[12] Sweeney KD, Donaghy E, Henderson D (2024). Patients’ experiences of GP consultations following the introduction of the new GP contract in Scotland: a cross-sectional survey. Br J Gen Pract.

[13] Mercer SW, Reynolds WJ (2002). Empathy and quality of care. Br J Gen Pract.

[14] Nembhard IM, David G, Ezzeddine I (2023). A systematic review of research on empathy in health care. Health Serv Res.

[15] Reilly D, Mercer SW, Bikker AP, Harrison T (2007). Outcome related to impact on daily living: preliminary validation of the ORIDL instrument. BMC Health Serv Res.

[16] Mercer SW, Reilly D, Watt GCM (2002). The importance of empathy in the enablement of patients attending the Glasgow Homoeopathic Hospital. Br J Gen Pract.

[17] Mercer SW, Maxwell M, Heaney D, Watt GC (2004). The Consultation and Relational Empathy (CARE) Measure: development and preliminary validation and reliability of an empathy-based consultation process measure. Fam Pract.

[18] Howie JG, Heaney DJ, Maxwell M, Walker JJ (1998). A comparison of a patient enablement instrument (PEI) against two established satisfaction scales as an outcome measure of primary care consultations. Fam Pract.

[19] Scottish Government Scottish Index of Multiple Deprivation 2020.

[20] NHS Scotland Organisations: NHS Scotland Healthboards. https://www.scot.nhs.uk/organisations.

[21] Kroenke K, Spitzer RL, Williams JBW, Löwe B (2009). An ultra-brief screening scale for anxiety and depression: the PHQ-4. Psychosomatics.

[22] Mercer SW, Jani BD, Maxwell M (2012). Patient enablement requires physician empathy: a cross-sectional study of general practice consultations in areas of high and low socioeconomic deprivation in Scotland. BMC Fam Pract.

[23] Mercer SW, Higgins M, Bikker AM (2016). General practitioners’ empathy and health outcomes: a prospective observational study of consultations in areas of high and low deprivation. Ann Fam Med.

[24] Dambha-Miller H, Feldman AL, Kinmonth AL, Griffin SJ (2019). Association between primary care practitioner empathy and risk of cardiovascular events and all-cause mortality among patients with type 2 diabetes: a population-based prospective cohort study. Ann Fam Med.

[25] Howick J, Moscrop A, Mebius A (2018). Effects of empathic and positive communication in healthcare consultations: a systematic review and meta-analysis. J R Soc Med.

[26] Liu X, Sawada Y, Takizawa T (2007). Doctor–patient communication: a comparison between telemedicine consultation and face-to-face consultation. Intern Med.

[27] Budd G, Griffiths D, Howick J (2022). Empathy in patient-clinician interactions when using telecommunication: a rapid review of the evidence. PEC Innov.

[28] Jabour AM (2024). A comparative study of patient–physician empathy in telehealth and traditional in-person visits. Digit Health.

[29] Scottish Government (2024). Care in the digital age: delivery plan 2024 to 2025.

[30] NHS England (2025). Fit for the future: 10 year health plan for England. https://assets.publishing.service.gov.uk/media/68760ad755c4bd0544dcae33/fit-for-the-future-10-year-health-plan-for-england.pdf.

[31] Mercer SW, Watt GCM (2007). The inverse care law: clinical primary care encounters in deprived and affluent areas of Scotland. Ann Fam Med.

[32] Mercer SW, Fitzpatrick B, Gourlay G (2007). More time for complex consultations in a high-deprivation practice is associated with increased patient enablement. Br J Gen Pract.

